# Changing Patterns of Substance Use During the Coronavirus Pandemic: Self-Reported Use of Tobacco, Alcohol, Cannabis, and Other Drugs

**DOI:** 10.3389/fpsyt.2021.633551

**Published:** 2021-05-26

**Authors:** Annemieke Benschop, Floor van Bakkum, Judith Noijen

**Affiliations:** ^1^Centre of Expertise Urban Vitality, Faculty of Health, Amsterdam University of Applied Science, Amsterdam, Netherlands; ^2^Jellinek Prevention, Arkin, Amsterdam, Netherlands

**Keywords:** COVID-19, coronavirus, substance use, tobacco, alcohol, cannabis, drugs

## Abstract

As in many other countries worldwide, the coronavirus pandemic prompted the implementation of an “intelligent lockdown” in the spring of 2020 in the Netherlands, including the closure of nightlife venues and cancellation of festivals. Such restrictions and social distancing could particularly affect people who use alcohol or other drugs in recreational settings and give rise to new challenges and additional needs in the field of addiction prevention and care. To monitor changes in substance use and provide services with practical directions for tailored prevention, an anonymous web survey was set up, targeting a convenience sample aged 16 years or older through various social media and other online channels. Between May and October 2020, a total of 6,070 participants completed the survey, mainly adolescents and young adults (16–24 years old). These data were used to explore and describe changing patterns in substance use. Overall results showed declined current use compared to “pre-corona,” but mask underlying variation in changing patterns, including discontinued (tobacco 10.4%, alcohol 11.3%, cannabis 16.3%, other drugs 30.4%), decreased (tobacco 23.0%, alcohol 29.1%, cannabis 17.4%, other drugs 20.7%), unchanged (tobacco 30.3%, alcohol 21.2%, cannabis 22.3%, other drugs 17.3%), increased (tobacco 29.6%, alcohol 32.1%, cannabis 32.9%, other drugs 25.3%), and (re)commenced use (tobacco 6.7%, alcohol 6.3%, cannabis 11.1%, other drugs 6.2%). Especially the use of drugs like ecstasy and nitrous oxide was discontinued or decreased due to the lack of social occasions for use. Increased use was associated with coping motives for all substance types. As measures combatting the coronavirus may need to be practiced for some time to come, possibly leading to prolonged changes in substance use with lingering “post-corona” consequences, timely and ongoing monitoring of changing patterns of substance use is vital for informing prevention services within this field.

## Introduction

The pandemic of coronavirus disease 2019 (COVID-19) has massively affected the lives of people all over the world. Countries have taken drastic measures to contain the outbreak, from curfews to national quarantines. In March 2020, the Dutch government implemented a so-called “intelligent lockdown” to mitigate the spread of the virus. Daycare centers, schools and universities were closed, as were sports clubs, libraries, cinemas, theaters, museums, restaurants and nightlife venues; large social gatherings and events were canceled; almost all “contact professions” (e.g., hairdressers, driving instructors, physiotherapists) were suspended; both public and private meetings of people from different households were rigorously restricted; and 1.5-m social distancing and work-from-home orders were issued. In June most measures were lifted or relaxed (provided 1.5-m distance was maintained, thus restricting numbers of guests and customers), though festivals and club nights remained prohibited. However, rising infection rates warranted gradually more stringent measures from August onwards, yet again impeding social occasions like sports games, cultural outings, going out for drinks or dinner, or inviting friends to a party at home.

The impacts of both the coronavirus and the measures taken to reduce its spread are severe and disrupting on many societal levels, including public mental health (1, 2). Several authors have predicted or expressed concern about increased substance use liability due to emotional distress (3–9). However, Rehm et al. (10) postulated two (not mutually exclusive) scenarios with opposite predictions regarding the impact of the current pandemic on the level and patterns of alcohol consumption. The first scenario predicts an increase in consumption due to psychological distress, while the second scenario predicts a lowered level of consumption due to decreased physical and financial availability.

There is a growing amount of literature about the coronavirus and substance use, but many of these studies address the heightened risks of people using substances in contracting the virus or having poorer disease prognosis [cf. (11)]. Research into changing patterns of substance use is less common and often limited to alcohol and/or tobacco (12–24); some studies (also) look into changes in the use of cannabis (25–30), but few were found that included other drugs like “party drugs” that tend to be predominantly used in social contexts affected by the coronavirus measures (31–33).

Measures combatting the coronavirus may need to be practiced until 2022 (34), possibly leading to prolonged changes in substance use with lingering “post-corona” consequences. Health policy makers and services are expected to proactively address the emerging changes and related risks needs. Monitoring changing patterns of substance use is therefore vital for prevention and addiction care when developing and delivering appropriate public health responses and interventions. With all festivals being suspended and nightlife venues closed due to the “intelligent lockdown,” prevention practice lost sight of a large and important group of people who use alcohol and other drugs in recreational settings. At the same time, restrictions and social distancing could particularly affect this population, resulting in changing substance use patterns and practices with associated risks. A signaling tool was rapidly needed to provide prevention services with practical directions for relevant and tailored educational information to promote healthy behaviors within this field.

Antenna Amsterdam (35) is an ongoing monitoring scheme that has been documenting developments and trends in recreational substance use in the Dutch capital since 1993, making it the oldest of such monitors running in Europe (36). Part of the mixed-methods approach is an annual on-site survey among varying target groups, including pub-goers and visitors of clubs and dance events. To address the needs of prevention services throughout the Netherlands for timely directions for targeted action an alternative nationwide online survey was set up. Interim national and regional results of the survey were regularly shared in dashboards and infographics within the Dutch network of prevention organizations to monitor changes in substance use patterns.

This paper is based on partial and preliminary data from this survey. Since the survey cannot be used to estimate drug use prevalence of the general population (37) and was not designed as an epidemiological effect study, the aim of this paper is not to test pre-formulated hypotheses about the impact of measures combating the coronavirus on public health, but to explore and describe changing patterns in substance use. Using the survey data we aim to assess to what extent the aforementioned scenarios of increased and decreased alcohol consumption (10) have taken place among people who use alcohol in the Netherlands, and if these scenarios also apply to the use of tobacco, cannabis and other drugs.

## Materials and Methods

### Sample

In May 2020, the “Antenne NL Corona Special” survey about substance use, gaming and gambling during the coronavirus pandemic went online. A convenience sample was recruited by circulating the link through the university and the network of organizations for treatment and prevention of substance use and abuse throughout the Netherlands. Methods included placing targeted advertisements on social media platforms such as Facebook and Instagram, posting messages on websites and in newsletters, and sharing via communication channels of various interventions and programs. There was no predefined target population and the link could be widely disseminated, but recruitment efforts could also be aimed at (varying) specific groups (e.g., students) or users (e.g., alcohol consumers). The questionnaire was accessible for anyone aged 16 years or older. By commencing the survey, participants gave electronic consent to understanding the study purpose, being aware of voluntariness and anonymity (no identifying information or IP address was recorded), and permitting storage and use of their responses.

Between 12 May and 13 October 2020, the survey was completed 6,380 times. Repeated participation was allowed and reported in 310 (4.9%) questionnaires. Because questions about the “pre-corona” period could be skipped when participating for the second or subsequent time, this paper is based on a selection of 6,070 questionnaires where participants indicated first-time participation (answered negatively to the first question “Have you participated in this survey before?”).

In this paper, we focus on the use of tobacco, alcohol, cannabis and other drugs (omitting gaming and gambling) prior to the time the measures combatting the coronavirus were enforced on 16 March 2020 in the Netherlands (further on: “pre-corona” use) and current use.

### Measures

The online self-report questionnaires included the following measures.

Demographics were covered by questions about sex (male, female, other), age, residential municipality, type of persons participants were living with (multiple choice: none, parents, partner, housemates, children, other), enrollment in school or university (no, secondary or secondary vocational school, higher professional school, or university), and current working situation.

For current substance use, as narrow a time frame as possible was chosen to take into account the rapidly changing corona situation. For alcohol, tobacco and cannabis this was the last week, but for other drugs that are usually not used weekly by most this was stretched to last month. In an effort to measure “typical” substance use prior to the corona pandemic for comparison, the same narrow time frames would not be appropriate. Instead, use of alcohol, tobacco and cannabis was asked retrospectively for the pre-corona month (15 Feb−15 Mar 2020) and use of other drugs was asked for the pre-corona year (15 Mar 2019–15 Mar 2020).

Use of tobacco, alcohol and cannabis was measured by questions about the number of use days per week (0–7) and average amount (number of cigarettes/glasses/joints) per use day. Use of other drugs was measured by a multiple choice list (yes, no) of eleven substances: ecstasy (XTC/MDMA), amphetamines, cocaine, nitrous oxide, ketamine, LSD, psychedelic mushrooms/truffles, GHB, 2C-B, 3-MMC/4-MMC and/or any other drug (excluding tobacco, alcohol, cannabis, and prescription drugs).

Changes in substance use were derived from weekly consumption for tobacco, alcohol and cannabis (see Analyses). For other drugs, participants were asked in a single overall question to self-indicate whether they were using (a lot) more or less (frequently) than “pre-corona.”

To asses motives for current use a short *ad-hoc* list of eight reasons was developed: Because I find it pleasant/fun/mind-expanding; Because I find it makes social moments more fun/cozy; Because I needed an outlet now that there are few other options; Because I wanted to feel less worried/afraid/angry/stressed; Because I wanted to feel less lonely; Because I couldn't resist, at a time when I actually didn't want to; Because I already had it at home; Because I always do at those moments, out of habit. Answer categories for each of these reasons were: totally agree, agree, neutral, disagree, totally disagree.

An *ad-hoc* eight-item multiple choice list (yes, no) was developed to asses reasons for current discontinued or decreased other drug use: It's better for my state of mind; It's better for my health/fitness; I had less free time; I had fewer social occasions (going out, appointments, visits, parties, etc.); I was home alone less often; Someone in my environment has asked for it; I was ill/did not feel well. This question was not asked for tobacco, alcohol or cannabis use.

### Analyses

For the purpose of analyses, age was recoded into four categories (16–17 years, 18–24 years, 25–39 years, and 40+ years) and residential municipality was recoded into two categories (large > 100.000 inhabitants, and small < 100.000 inhabitants). Three working situations were distinguished: not working [no job or own business or (most) work has come to standstill], working from home (mostly), and working on location (mostly). And five types of household were derived from type of persons participants were living with: alone, with partner/housemates only, with parents only (and any siblings), with children (and any partner or other persons), and other.

For tobacco, alcohol and cannabis prevalence rates were derived from number of use days per week. Responses of large amounts per day (sometimes up to hundreds) were not classified as invalid and deleted, but perceived as meaning “a lot” and maximized around the 97.5th percentile (80 cigarettes, 20 glasses and 20 joints). Days per week and amount per day were multiplied to derive weekly consumption. Number of other drug types used was derived by counting the number of positive answers to the multiple choice questions, excluding the “other drug” category.

After recoded and derived variables were created, a four-step analyses procedure was carried out.

First, for each type of substance a selection was made of respondents with either current use or “pre-corona” use. Descriptive statistics were calculated for the total sample and the subsamples of selected respondents (Table 1).

**Table 1:** (Sub)sample characteristics.

	**Total sample**	**Subsamples with “pre-corona” and/or current use**^**a**^			
		**Tobacco**	**Alcohol**	**Cannabis**	**Other drugs^**b**^**
*n*	6,070	3,310	5,176	2,956	3,072
% of total sample	–	54.5%	85.3%	48.7%	50.6%
**Sex**					
Male	50.0%	56.4%	50.6%	62.4%	58.5%
Female	49.5%	43.0%	48.9%	36.9%	41.0%
Other	0.5%	0.6%	0.5%	0.7%	0.5%
**Age**					
Average (SD)	29.1 (16.8)	23.4 (11.8)	28.1 (16.0)	20.5 (7.3)	21.9 (8.0)
16–17	20.7%	26.4%	20.2%	30.5%	20.0%
18–24	43.7%	53.5%	46.5%	59.0%	61.8%
25–39	12.9%	10.5%	13.2%	7.6%	13.8%
40+	22.7%	9.5%	20.2%	2.9%	4.4%
**Place of residence**					
Small (pop. < 100.000)	52.2%	53.0%	51.7%	51.6%	47.1%
Large (pop. > 100.000)	47.8%	47.0%	48.3%	48.4%	52.9%
**Student**					
No	41.1%	30.1%	39.1%	21.5%	28.9%
Secondary (vocational)	34.4%	44.0%	34.1%	48.7%	36.9%
Higher professional or university	24.4%	25.9%	26.7%	29.8%	34.2%
**Work**					
Not working	38.6%	39.2%	37.7%	40.4%	38.0%
Working from home	15.1%	9.7%	15.3%	7.2%	11.0%
Working on location	46.4%	51.1%	47.1%	52.4%	51.0%
**Household**					
Alone	12.3%	9.6%	11.9%	7.0%	9.0%
With partner/housemate(s)	25.5%	21.4%	25.6%	19.5%	26.0%
With parent(s)	48.4%	59.7%	49.5%	67.3%	57.3%
With child(ren)	11.4%	6.3%	10.7%	3.5%	4.6%
Other	2.4%	2.9%	2.4%	2.7%	3.1%

a *selection of respondents with either “pre-corona” use (“pre-corona” month, 15 Feb−15 Mar 2020, for tobacco, alcohol and cannabis; “pre-corona” year, 15 Mar 2019–15 Mar 2020, for other drugs) or current use (last week for tobacco, alcohol and cannabis; last month for other drugs)*.

b *ecstasy, amphetamines, cocaine, nitrous oxide, ketamine, LSD, psychedelic mushrooms/truffles, GHB, 2C-B, 3-MMC/4-MMC, and/or any other drug (excluding tobacco, alcohol, cannabis, and prescription drugs)*.

Second, within the subsamples, “pre-corona” and current use were compared using McNemar tests for prevalence rates, and paired *T-*tests for average number of days, average amount per day and average weekly consumption (Table 2).

**Table 2:** “Pre-corona” and current use of tobacco, alcohol, cannabis, and other drugs (paired tests).

	**“Pre-corona” use**	**Current use**	**ChiSq/T (df)**	***p***	**Cohen's g/d**
**Tobacco (*****n=*** **3,310)**^**a**^					
Prevalence rate	93.3%	89.6%	26.343	<0.001	0.109
Average days per week (SD)^b^	5.4 (2.2)	5.6 (2.1)	5.593 (2,744)	<0.001	0.107
Average amount (cigarettes) per day (SD)^b^	12.7 (15.9)	12.0 (14.4)	−3.862 (2,744)	<0.001	−0.074
Average weekly consumption (SD)^b^	79.9 (109.2)	77.6 (100.9)	−1.901 (2,744)	0.057	−0.036
**Alcohol (*****n=*** **5,176)**^**a**^					
Prevalence rate	93.7%	88.7%	72.422	<0.001	0.141
Average days per week (SD)^b^	2.9 (1.8)	3.2 (1.9)	12.675 (4,263)	<0.001	0.194
Average amount (glasses) per day (SD)^b^	5.7 (4.6)	5.1 (4.2)	−10.983 (4,263)	<0.001	−0.168
Average weekly consumption (SD)^b^	17.2 (20.7)	17.3 (20.1)	−0.556 (4,263)	0.578	0.009
**Cannabis (*****n=*** **2,956)**^**a**^					
Prevalence rate	88.9%	83.7%	29.660	<0.001	0.096
Average days per week (SD)^b^	4.3 (2.4)	4.8 (2.8)	12.110 (2,145)	<0.001	0.261
Average amount (joints) per day (SD)^b^	3.8 (4.5)	3.9 (4.1)	1.158 (2,145)	0.247	0.025
Average weekly consumption (SD)^b^	20.8 (31.7)	22.3 (29.4)	3.332 (2,145)	0.001	0.072
**Other drugs (*****n=*** **3,072)**^**a**^, **^c^**					
Any other drugs	93.8%	69.6%	492.032	<0.001	0.331
Ecstasy	73.2%	38.2%	794.952	<0.001	0.370
Amphetamines	32.9%	15.6%	386.663	<0.001	0.367
Cocaine	39.5%	24.3%	283.323	<0.001	0.304
Nitrous oxide	47.1%	20.7%	604.881	<0.001	0.374
Ketamine	35.6%	24.0%	179.259	<0.001	0.252
LSD	8.8%	6.0%	30.754	<0.001	0.188
Psychedelic mushrooms/truffles	14.6%	9.8%	49.920	<0.001	0.172
GHB	7.3%	3.4%	84.211	<0.001	0.354
2C-B	28.8%	18.2%	135.697	<0.001	0.208
3-MMC/4-MMC	10.1%	8.8%	6.500	0.011	0.085
Average number of drug types (SD)^b^	3.7 (2.2)	2.6 (1.8)	−25.103 (1,946)	<0.001	−0.569

a *subsamples of respondents with either “pre-corona” use (“pre-corona” month, 15 Feb−15 Mar 2020, for tobacco, alcohol and cannabis; “pre-corona” year, 15 Mar 2019–15 Mar 2020, for other drugs) or current use (last week for tobacco, alcohol and cannabis; last month for other drugs)*.

b *applies only to those with “pre-corona” use and current use, respectively*.

c *ecstasy, amphetamines, cocaine, nitrous oxide, ketamine, LSD, psychedelic mushrooms/truffles, GHB, 2C-B, 3-MMC/4-MMC, and/or any other drug (excluding tobacco, alcohol, cannabis, and prescription drugs)*.

Third, five groups were identified within each subsample, based on the difference between “pre-corona” and current use: (1) Stopped: “Pre-corona” use, but no current use; (2) Less: Both “pre-corona” and current use, and lower weekly consumption of tobacco/alcohol/cannabis or reported (a lot) less (frequent) use of other drugs; (3) Same: Both “pre-corona” and current use, and the same weekly consumption of tobacco/alcohol/cannabis or reported the same use of other drugs; (4) More: Both “pre-corona” and current use, and higher weekly consumption of tobacco/alcohol/cannabis or reported (a lot) more (frequent) use of other drugs; and (5) Started: Current use, but no “pre-corona” use. “Pre-corona” and current use were compared across these five groups using ChiSq and ANOVA tests (Tables 3A,B).

**Table 3A:** Change in the use of tobacco, alcohol and cannabis—current use compared to “pre-corona” use.

	**Stopped^**a**^**	**Less^**b**^**	**Same^**c**^**	**More^**d**^**	**Started^**e**^**	**ChiSq/F(df = 3)**	***p***	**EtaSq**
**Tobacco (*****n=*** **3,310)**^**f**^
% (*n*)	10.4% (344)	23.0% (762)	30.3% (1, 3)	29.6% (980)	6.7% (221)			
Av. “pre-corona” use (SD)					N/A			
Days per week	3.2 (2.4)	5.8 (1.8)	6.1 (1.9)	4.5 (2.4)		221,638	<0.001	0.177
Amount per day	6.3 (8.5)	15.6 (18.8)	15.5 (17.1)	4.6 (9.7)		80,361	<0.001	0.072
Weekly consumption	28.1 (44.0)	97.8 (127.1)	104.8 (121.0)	40.7 (59.0)		105,967	<0.001	0.093
Av. current use (SD)	N/A							
Days per week		4.6 (2.4)	6.1 (1.9)	5.9 (1.6)	2.7 (2.1)	242,571	<0.001	0.197
Amount per day		7.6 (1.2)	15.5 (17.1)	11.9 (13.0)	4.6 (7.7)	68,801	<0.001	0.065
Weekly consumption		42.8 (67.7)	104.8 (121.0)	76.8 (91.0)	20.4 (52.4)	85,848	<0.001	0.080
Change in weekly consumption			N/A					
2 cigarettes or less	31.7%	7.2%		6.1%	41.2%			
2–10 cigarettes	26.7%	18.4%		22.0%	29.9%			
10–20 cigarettes	10.8%	18.8%		19.1%	9.5%			
More than 20 cigarettes	30.8%	55.6%		52.8%	19.5%			
**Alcohol (*****n=*** **5,176)**^**f**^
% (*n*)	11.3% (585)	29.1% (1,505)	21.2% (1,098)	32.1% (1,661)	6.3% (327)			
Av. “pre-corona” use (SD)					N/A			
Days per week	2.3 (1.5)	3.3 (1.7)	3.1 (2.2)	2.3 (1.5)		135,471	<0.001	0.077
Amount per day	4.6 (4.0)	7.4 (4.9)	5.0 (4.6)	4.7 (3.9)		127,145	<0.001	0.073
Weekly consumption	10.4 (15.2)	24.6 (24.0)	16.8 (23.2)	10.7 (11.6)		158,487	<0.001	0.089
Av. current use (SD)	N/A							
Days per week		2.3 (1.5)	3.2 (2.2)	4.0 (1.7)	2.0 (1.3)	328,314	<0.001	0.177
Amount per day		4.3 (3.4)	4.9 (4.6)	6.0 (4.4)	4.0 (3.8)	57,653	<0.001	0.036
Weekly consumption		10.0 (11.2)	16.8 (23.2)	24.4 (21.7)	9.2 (15.5)	172,304	<0.001	0.101
Change in weekly consumption			N/A					
2 glasses or less	26.7%	15.8%		13.1%	33.6%			
2–10 glasses	45.0%	41.9%		44.7%	43.7%			
10–20 glasses	17.3%	21.8%		23.5%	14.1%			
More than 20 glasses	11.1%	20.5%		18.8%	8.6%			
**Cannabis (*****n=*** **2,956)**^**f**^
% (*n*)	16.3% (483)	17.4% (514)	22.3% (659)	32.9% (973)	11.1% (327)			
Av. “pre-corona” use (SD)					N/A			
Days per week	2.1 (1.8)	5.2 (1.9)	4.8 (2.6)	3.4 (2.2)		229,751	<0.001	0.208
Amount per day	1.7 (1.9)	5.7 (5.4)	4.4 (5.4)	2.3 (2.2)		123,249	<0.001	0.123
Weekly consumption	5.2 (11.7)	33.4 (38.1)	27.5 (39.1)	9.5 (13.4)		146,007	<0.001	0.143
Av. current use (SD)	N/A							
Days per week		3.8 (2.3)	4.8 (2.6)	5.3 (1.8)	2.2 (1.7)	182,895	<0.001	0.182
Amount per day		3.0 (3.0)	4.4 (5.4)	3.9 (3.4)	1.7 (1.6)	45,216	<0.001	0.052
Weekly consumption		14.5 (20.9)	27.5 (39.1)	22.8 (24.3)	5.0 (10.4)	60,038	<0.001	0.068
Change in weekly consumption			N/A					
2 joints or less	67.1%	17.5%		20.3%	60.6%			
2–10 joints	21.7%	36.8%		36.1%	29.4%			
10–20 joints	4.6%	19.5%		24.5%	5.5%			
More than 20 joints	6.6%	26.3%		19.1%	4.6%			

**Table 3B:** Change in the use of other drugs—current use compared to “pre-corona” use.

	**Stopped^**a**^**	**Less^**b**^**	**Same^**c**^**	**More^**d**^**	**Started^**e**^**	**ChiSq/F(df = 3)**	***p***	**Cramer's V/EtaSq**
**Other drugs (*****n=*** **3,072)**^**f**^, **^g^**
% (*n*)	30.4% (935)	20.7% (637)	17.3% (532)	25.3% (778)	6.2% (190)			
“Pre-corona” use					N/A			
Ecstasy	68.2%	89.0%	80.6%	78.9%		99.507	<0.001	0.186
Amphetamines	22.0%	46.2%	39.1%	38.7%		112.514	<0.001	0.198
Cocaine	30.4%	51.0%	46.8%	45.8%		82.622	<0.001	0.169
Nitrous oxide	44.4%	54.0%	52.8%	52.2%		19.018	<0.001	0.081
Ketamine	21.0%	52.1%	43.2%	43.2%		184.631	<0.001	0.253
LSD	4.5%	13.8%	9.4%	11.4%		45.095	<0.001	0.125
Psychedelic mushrooms/truffles	11.0%	17.0%	22.0%	15.6%		32.356	<0.001	0.106
GHB	4.2%	11.3%	8.3%	9.0%		29.680	<0.001	0.101
2C-B	18.7%	42.5%	33.3%	33.8%		110.196	<0.001	0.196
3-MMC/4-MMC	4.9%	13.8%	10.3%	15.4%		57.302	<0.001	0.141
Av. number of drug types (SD)	2.3 (1.6)	4.0 (2.1)	3.5 (2.1)	3.5 (2.2)		102.731	<0.001	0.097
Current use	N/A							
Ecstasy		43.3%	55.1%	65.4%	50.0%	71.099	<0.001	0.182
Amphetamines		17.7%	20.7%	30.2%	11.6%	48.839	<0.001	0.151
Cocaine		29.4%	35.5%	43.4%	16.8%	60.973	<0.001	0.169
Nitrous oxide		22.6%	28.0%	36.0%	33.2%	31.871	<0.001	0.122
Ketamine		29.7%	31.8%	45.5%	13.7%	86.392	<0.001	0.201
LSD		7.8%	9.4%	10.4%	2.1%	14.237	0.003	0.082
Psychedelic mushrooms/truffles		10.2%	16.0%	14.9%	18.9%	13.611	0.003	0.080
GHB		3.5%	5.1%	6.6%	2.1%	10.718	0.013	0.071
2C-B		19.8%	21.2%	36.4%	18.9%	67.312	<0.001	0.177
3-MMC/4-MMC		8.3%	10.3%	18.9%	7.4%	45.818	<0.001	0.146
Av. number of drug types (SD)		2.0 (1.4)	2.4 (1.6)	3.2 (1.9)	1.8 (1.3)	75.540	<0.001	0.096

a *“Pre-corona” use, but no current use*.

b *both “pre-corona” and current use, and lower weekly consumption of tobacco/alcohol/cannabis or reported (a lot) less (frequent) use of other drugs*.

c *both “pre-corona” and current use, and the same weekly consumption of tobacco/alcohol/cannabis or reported the same use of other drugs*.

d *both “pre-corona” and current use, and higher weekly consumption of tobacco/alcohol/cannabis or reported (a lot) more (frequent) use of other drugs*.

e *current use, but no “pre-corona” use*.

f *subsamples of respondents with either “pre-corona” use (“pre-corona” month, 15 Feb−15 Mar 2020, for tobacco, alcohol, and cannabis; “pre-corona” year, 15 Mar 2019–15 Mar 2020, for other drugs) or current use (last week for tobacco, alcohol and cannabis; last month for other drugs)*.

g *ecstasy, amphetamines, cocaine, nitrous oxide, ketamine, LSD, psychedelic mushrooms/truffles, GHB, 2C-B, 3-MMC/4-MMC, and/or any other drug (excluding tobacco, alcohol, cannabis, and prescription drugs)*.

Fourth, associations between change in use and demographic characteristics (Supplementary Tables 1–4), reasons for current use (Table 4) and reasons for discontinued/decreased use (Table 5) were examined using ChiSq and ANOVA tests. When comparing demographics, the “other” category for sex and household were omitted from analyses. When comparing reasons for current use, respondents without current use (“stopped”) were omitted from analyses. The latter analyses were limited to respondents with discontinued (“stopped”) or decreased (“less”) use of other drugs.

**Table 4:** Reasons for current use^a^.

	**Total^**b**^**	**Less^**c**^**	**Same^**d**^**	**More^**e**^**	**Started^**f**^**	**F (df = 3)**	***p***	**EtaSq**
Tobacco^(n)^	2,966	762	1,003	980	221			
Because I find it pleasant/fun/mind-expanding	0.5 (1.1)	0.5 (1.1)	0.5 (1.1)	0.5 (1.1)	0.1 (1.3)	8.187	<0.001	0.008
Because I find it makes social moments more fun/cozy	0.6 (1.1)	0.8 (1.0)	0.5 (1.2)	0.7 (1.1)	0.4 (1.3)	13.336	<0.001	0.013
Because I needed an outlet now that there are few other options	0.0 (1.3)	−0.1 (1.3)	−0.2 (1.3)	0.3 (1.3)	−0.1 (1.4)	29.245	<0.001	0.029
Because I wanted to feel less worried/afraid/angry/stressed	−0.1 (1.4)	−0.1 (1.4)	−0.3 (1.4)	0.1 (1.4)	−0.3 (1.5)	13.688	<0.001	0.014
Because I wanted to feel less lonely	−0.8 (1.2)	−0.9 (1.1)	−0.9 (1.2)	−0.7 (1.2)	−0.9 (1.2)	8.643	<0.001	0.009
Because I couldn't resist, at a time when I actually didn't want to	−0.4 (1.3)	−0.4 (1.3)	−0.5 (1.3)	−0.2 (1.3)	−0.5 (1.4)	11.234	<0.001	0.011
Because I already had it at home	0.0 (1.3)	0.0 (1.3)	0.0 (1.3)	0.2 (1.3)	−0.4 (1.4)	16.605	<0.001	0.017
Because I always do at those moments, out of habit	0.6 (1.2)	0.6 (1.2)	0.9 (1.1)	0.6 (1.2)	−0.6 (1.3)	111.530	<0.001	0.101
Alcohol^(n)^	4,591	1,505	1,098	1,661	327			
Because I find it pleasant/fun/mind-expanding	1.0 (1.0)	1.0 (1.0)	0.9 (1.0)	1.1 (0.9)	0.6 (1.2)	21.349	<0.001	0.014
Because I find it makes social moments more fun/cozy	0.9 (1.1)	1.0 (1.0)	0.7 (1.1)	1.0 (1.0)	0.6 (1.3)	26.893	<0.001	0.017
Because I needed an outlet now that there are few other options	−0.4 (1.3)	−0.6 (1.3)	−0.7 (1.3)	0.0 (1.4)	−0.7 (1.3)	71.275	<0.001	0.045
Because I wanted to feel less worried/afraid/angry/stressed	−0.8 (1.3)	−0.9 (1.2)	−1.0 (1.2)	−0.6 (1.3)	−0.9 (1.3)	32.787	<0.001	0.021
Because I wanted to feel less lonely	−1.0 (1.2)	−1.0 (1.2)	−1.1 (1.1)	−0.8 (1.3)	−1.1 (1.2)	22.367	<0.001	0.014
Because I couldn't resist, at a time when I actually didn't want to	−1.1 (1.1)	−1.2 (1.0)	−1.2 (1.1)	−0.8 (1.2)	−1.3 (1.1)	40.906	<0.001	0.026
Because I already had it at home	−0.3 (1.3)	−0.3 (1.3)	−0.5 (1.3)	−0.1 (1.3)	−0.5 (1.4)	27.254	<0.001	0.018
Because I always do at those moments, out of habit	−0.1 (1.3)	−0.2 (1.3)	0.0 (1.3)	−0.1 (1.3)	−1.0 (1.2)	58.511	<0.001	0.037
Cannabis^(n)^	2,473	514	659	973	327			
Because I find it pleasant/fun/mind-expanding	1.5 (0.7)	1.5 (0.7)	1.5 (0.8)	1.5 (0.7)	1.3 (0.8)	11.109	<0.001	0.013
Because I find it makes social moments more fun/cozy	0.8 (1.1)	1.0 (1.1)	0.7 (1.2)	0.9 (1.1)	0.4 (1.2)	19.254	<0.001	0.023
Because I needed an outlet now that there are few other options	0.2 (1.4)	0.1 (1.3)	0.1 (1.4)	0.5 (1.3)	0.0 (1.5)	17.827	<0.001	0.021
Because I wanted to feel less worried/afraid/angry/stressed	0.1 (1.5)	0.1 (1.5)	0.0 (1.5)	0.2 (1.4)	−0.2 (1.5)	6.498	<0.001	0.008
Because I wanted to feel less lonely	−0.6 (1.4)	−0.5 (1.4)	−0.7 (1.4)	−0.4 (1.4)	−0.7 (1.4)	5.442	0.001	0.007
Because I couldn't resist, at a time when I actually didn't want to	−0.5 (1.4)	−0.5 (1.3)	−0.7 (1.4)	−0.3 (1.4)	−1.0 (1.3)	22.442	<0.001	0.027
Because I already had it at home	0.1 (1.4)	0.2 (1.3)	0.1 (1.4)	0.4 (1.3)	−0.5 (1.4)	39.827	<0.001	0.046
Because I always do at those moments, out of habit	0.2 (1.4)	0.4 (1.3)	0.4 (1.4)	0.3 (1.3)	−1.1 (1.1)	111.739	<0.001	0.120
Other drugs^(n)^	2,137	637	532	778	190			
Because I find it pleasant/fun/mind-expanding	1.4 (0.9)	1.3 (0.9)	1.4 (0.9)	1.5 (0.8)	1.2 (1.1)	8.975	<0.001	0.012
Because I find it makes social moments more fun/cozy	0.8 (1.1)	0.7 (1.1)	0.7 (1.1)	0.9 (1.0)	0.5 (1.3)	12.056	<0.001	0.017
Because I needed an outlet now that there are few other options	−0.1 (1.4)	−0.2 (1.4)	−0.3 (1.4)	0.3 (1.5)	−0.3 (1.5)	23.497	<0.001	0.032
Because I wanted to feel less worried/afraid/angry/stressed	−0.7 (1.4)	−1.0 (1.2)	−1 (1.2)	−0.5 (1.4)	−0.5 (1.5)	20.810	<0.001	0.028
Because I wanted to feel less lonely	−1.0 (1.3)	−1.1 (1.1)	−1.2 (1.1)	−0.7 (1.4)	−0.8 (1.4)	22.096	<0.001	0.030
Because I couldn't resist, at a time when I actually didn't want to	−0.9 (1.3)	−1.0 (1.2)	−1.0 (1.2)	−0.6 (1.4)	−0.9 (1.3)	22.505	<0.001	0.031
Because I already had it at home	−0.5 (1.4)	−0.6 (1.3)	−0.7 (1.3)	−0.2 (1.4)	−0.5 (1.4)	15.996	<0.001	0.022
Because I always do at those moments, out of habit	−0.9 (1.2)	−1 (1.1)	−0.9 (1.2)	−0.7 (1.3)	−1.2 (1.1)	13.125	<0.001	0.018

a *average (SD) score on Likert scale: totally agree (+2), agree (+1), neutral (0), disagree (−1), totally disagree (−2). Applies only to respondents with current use*.

b *subsamples of respondents with current use (last week for tobacco, alcohol and cannabis; last month for other drugs)*.

c *both “pre-corona” and current use, and lower weekly consumption of tobacco/alcohol/cannabis or reported (a lot) less (frequent) use of other drugs*.

d *both “pre-corona” and current use, and the same weekly consumption of tobacco/alcohol/cannabis or reported the same use of other drugs*.

e *both “pre-corona” and current use, and higher weekly consumption of tobacco/alcohol/cannabis or reported (a lot) more (frequent) use of other drugs*.

f *current use, but no “pre-corona” use*.

*ecstasy, amphetamines, cocaine, nitrous oxide, ketamine, LSD, psychedelic mushrooms/truffles, GHB, 2C-B, 3-MMC/4-MMC, and/or any other drug (excluding tobacco, alcohol, cannabis, and prescription drugs)*.

**Table 5:** Reasons for discontinued/decreased other drug use.

	**Total^**a**^**	**Stopped^**b**^**	**Less^**c**^**	**ChiSq (df = 1)**	***p***	**Cramer's V**
Other drugs (n)^d^	1,572	935	637			
It's better for my state of mind	19.3%	19.6%	19.0%	0.081	0.776	0.007
It's better for my health/fitness	26.1%	25.9%	26.4%	0.047	0.828	0.005
I had less free time	9.9%	8.9%	11.5%	2.828	0.093	0.042
I had fewer social occasions (going out, appointments, visits, parties, etc.)	65.3%	60.5%	72.2%	22.796	<0.001	0.120
I was home alone less often	5.3%	4.8%	6.1%	1.285	0.257	0.029
Someone in my environment has asked for it	3.1%	3.6%	2.4%	2.061	0.151	0.036
I was ill/did not feel well	2.4%	1.8%	3.1%	2.879	0.090	0.043

a *subsamples of respondents with discontinued (“stopped”) or decreased (“less”) other drug use (last month use compared to “pre-corona” year, 15 Mar 2019–15 Mar 2020)*.

b *“Pre-corona” use, but no current use*.

c *both “pre-corona” and current use, and lower weekly consumption of tobacco/alcohol/cannabis or reported (a lot) less (frequent) use of other drugs*.

d *ecstasy, amphetamines, cocaine, nitrous oxide, ketamine, LSD, psychedelic mushrooms/truffles, GHB, 2C-B, 3-MMC/4-MMC, and/or any other drug (excluding tobacco, alcohol, cannabis, and prescription drugs)*.

Overall model results are presented (no pairwise *post-hoc* tests were computed). Analyses were carried out using IBM SPSS Statistics 25.

## Results

Table 1 shows numbers and characteristics of the total sample and subsamples of respondents with “pre-corona” and/or current use of tobacco, alcohol, cannabis and other drugs. The majority of respondents use alcohol; the subsamples of those using tobacco, cannabis and other drugs comprise about half of the total sample. In the total sample males and females, and respondents from both small and large municipalities are equally divided, while males make up a (small) majority in the subsamples. Both the total sample and subsamples consist for a large part of young adults (18–24 years) and students living with parents. This is especially true for subsamples of respondents who use tobacco, cannabis and other drugs; a little less so for the subsample of respondents who use alcohol. As far as respondents have a job or own a business, they mostly work on location and not from home.

Within all subsamples current prevalence rates are lower compared to “pre-corona” (Table 2). Those who indicated continued use of tobacco and alcohol consumed increased in frequency (tobacco: from 5.4 to 5.6 days per week; alcohol from 2.9 to 3.2 days per week), but decreased in amounts (tobacco: from 12.7 to 12.0 cigarettes per day; alcohol: from 5.7 to 5.1 glasses per day), so that the average weekly consumption remained the same. Those continuing to use cannabis also increased their frequency of use (from 4.3 to 4.8 days per week), but did not change the amount. Average weekly consumption of cannabis therefore increased from 20.8 to 22.3 joints per week. Within the category of other drugs, ecstasy and nitrous oxide showed the most prominent decline in use. Respondents with both “pre-corona” and current drug use narrowed their drugs palette and used fewer different types of drugs (from 3.7 to 2.6 drug types on average).

While current overall prevalence rates in the subsamples were either lower than or similar to “pre-corona,” Tables 3A,B show that there are also respondents with increased use, including those who did not use in the “pre-corona” period but currently do. The latter group (“started”) formed around 6% of the subsamples of respondents who used tobacco, alcohol and other drugs, and 11.1% for cannabis.

Respondents who started using tobacco, alcohol and cannabis since the coronavirus measures came into effect do so less frequently and in smaller amounts than those already using (Table 3A). Almost a third of respondents using tobacco (29.6%), alcohol (32.1%), and cannabis (32.9%) smoked and drank more than “pre-corona” (“more”) and increased both frequency and amount of use, amounting to about a doubling of the weekly consumption. In some cases the total increase is limited to 2 cigarettes/glasses/joints per week, but there are also those who show a substantial increase in weekly consumption of more than 20 cigarettes/glasses/joints. Conversely, respondents using less tobacco (23.0%), alcohol (29.1%), and cannabis (17.4%) (“less”) reduced both frequency and quantity, cutting the average weekly consumption in half. Notably, these respondents with decreased use show the highest “pre-corona” weekly consumption of alcohol (average 24.6 glasses) and cannabis (average 33.4 joints), and the second highest weekly tobacco consumption (average 97.8 cigarettes). Those who stopped using tobacco (10.4%), alcohol (11.3%), and cannabis (16.3%) since the coronavirus measures came into effect (“stopped”) showed less extensive “pre-corona” consumption patterns. Current consumption of tobacco and cannabis was highest among respondents with unchanged use (“same”); current alcohol use was heaviest among those with increased use (“more”).

Compared to tobacco, alcohol and cannabis, a larger proportion of respondents stopped using other drugs (“stopped” 30.4%) (Table 3B). These respondents showed a less extensive pattern of “pre-corona” use compared to respondents with continued use (2.3 compared to 3.5–4.0 drug types on average). In fact, many used no more than one type of drug before the coronavirus measures came into effect, mostly ecstasy or nitrous oxide. Respondents reporting decreased (but continued) other drug use (“less”) reduced the number of drug types used from 4.0 to 2.0 on average [paired T(df) = −27.020(636), *p* <0.001, Cohen's d = −1.071]. Markedly, respondents reporting increased use (“more”) also showed a reduction in the number of drug types used [from 3.5 to 3.2, T(df) = −5.259(777), *p* ≤ 0.001, Cohen's d = −0.189]. Moreover, current prevalence rates were lower than “pre-corona” rates for ecstasy (65.4% vs. 78.9%, McNemar paired ChiSq = 45.255, *p* ≤ 0.001, Cohen's g = 0.220), amphetamines (30.2 vs. 38.7%, ChiSq = 24.006, *p* ≤ 0.001, Cohen's g = 0.188), nitrous oxide (36.0 vs. 52.2%, ChiSq = 73.703, *p* ≤ 0.001, Cohen's g = 0.297) and GHB (6.6 vs. 9.0%, ChiSq = 7.200, *p* = 0.007, Cohen's g = 0.211), and only higher for 3-MMC/4-MMC (18.9 vs. 15.4%, ChiSq = 8.557, *p* = 0.003, Cohen's g = 0.171).

Associations between change in use and demographic characteristics varied between types of substance. For alcohol, increased use was relatively more common among adults (25–39 years) and decreased use relatively more common among young adults (18–24 years). For other drugs, however, the opposite was true. Supplementary Material about demographic characteristics associated with changing patterns in substance use is available for professionals seeking input for tailored prevention.

Regardless of change in substances use, the most endorsed reason for current use of alcohol, cannabis or other drugs was either “Because I find it pleasant/fun/mind-expanding” or “Because I find it makes social moments more fun/cozy” (Table 5). Tobacco was often used out of habit. On face value, this seemed especially true for those with unchanged use (“same” average score 0.9, compared to −0.6 to 0.6 in other four groups). Respondents with unchanged use of alcohol also seemed to report habitual use more often than the other groups (0.0, compared to −0.1 to −1.0). Those with increased use of tobacco, alcohol, cannabis or other drugs (“more”) showed relatively high scores for the other reasons of use (“I needed an outlet…”, “I wanted to feel less worried…”, “I wanted to feel less lonely,” “I couldn't resist…”, and “I already had it at home”).

Having fewer social occasions than “pre-corona” was the most important reason to discontinue or decrease other drug use (65.3%), followed by physical (26.1%), and mental (19.3%) health. Overall, those who reduced their use of other drugs and those who had stopped using altogether reported similar reasons for doing so, but lack of social occasions was endorsed more often by respondents with decreased other drug use.

## Discussion

This paper is based on data from a survey about “pre-corona” (before measures combatting the coronavirus pandemic came into effect in March 2020) and current substance use among Dutch respondents aged 16 years and older recruited through online channels. The survey was set up as a monitoring tool, using a short questionnaire and a convenience sample, to provide descriptive results for prevention practice.

The total sample was divided into subsamples of 3,310 respondents who had smoked tobacco either during the “pre-corona” month or last week, 5,176 respondents who had drank alcohol, 2,956 respondents who had used cannabis, and 3,072 respondents who had used other drugs (e.g., ecstasy, amphetamines, cocaine, nitrous oxide) in the “pre-corona” year or last month. Within these subsamples, overall results showed declined use compared to “pre-corona.” However, overall figures mask underlying variation in changing patterns, including discontinued (tobacco 10.4%, alcohol 11.3%, cannabis 16.3%, other drugs 30.4%), decreased (tobacco 23.0%, alcohol 29.1%, cannabis 17.4%, other drugs 20.7%), unchanged (tobacco 30.3%, alcohol 21.2%, cannabis 22.3%, other drugs 17.3%), increased (tobacco 29.6%, alcohol 32.1%, cannabis 32.9%, other drugs 25.3%), and (re)commenced use (tobacco 6.7%, alcohol 6.3%, cannabis 11.1%, other drugs 6.2%). Others have also found both less and more substance use following enforcement of coronavirus measures (12–16, 20–27, 29, 38). The two opposite scenarios Rehm et al. (10) predicted from literature and a review of the effects of past economic crises on alcohol consumption, one with increased and one with decreased use, apparently co-exist and also pertain to other substances. These results inform prevention practice about differential effects of corona measures on substance use that are masked by population trend curves, as the effects of opposite patterns of increased and decreased use cancel each other out.

Discontinued use was found to be much more common for other drugs than for tobacco, alcohol and cannabis, but for all substance types applied that those who stopped using showed less extensive “pre-corona” consumption patterns. Notably, the groups with decreased use showed relatively high levels of “pre-corona” use. This finding contradicts other studies who reported heavier pre-pandemic drinking patterns among respondents with increased alcohol use (13, 20). In this study, increased use of any of the substances was not associated with heavier “pre-corona” use. Current consumption of tobacco and cannabis was highest for respondents with unchanged use, while respondents with increased use showed most extensive current use of alcohol and other drugs, although the latter group did show lower prevalence rates for ecstasy, amphetamines, nitrous oxide and GHB. Respondents having taken up substance use (again) after the coronavirus measures came into effect showed less extensive current consumption patterns compared to the other groups.

Associations between change in use and demographic characteristics varied between types of substance, indicating for instance that different age groups are at risk for increased use of alcohol and other drugs. This underlines the need for tailored prevention targeting specific populations for specific substances. Associations between change in use and reasons for current use showed a consistent pattern across different substances. All substances, regardless of change in use, were often used for pleasure and social reasons. In fact, the lack of social occasions was reported as the main reason for discontinued and decreased other drug use. But respondents with increased use of tobacco, alcohol, cannabis and other drugs were also more likely to report additional reasons for use, in particular needing an outlet and wanting to feel less worried/afraid/angry/stressed or lonely. These reasons can be seen as coping motives, which have been linked to problematic use of alcohol [e.g., (39)], cannabis [e.g., (40)], and ecstasy [e.g., (41)] in general, and have more specifically been found to mediate the link between stressors (having children at home, depression, social connectedness, income loss, and living alone) and alcohol-related problems during the coronavirus pandemic (42). This is perhaps the most important finding from a prevention point of view.

In this paper, we looked at changes in the use of tobacco, alcohol, cannabis and other drugs separately. Further analyses, taking into account combined use, should reveal whether the groups with increased or decreased use overlap, or whether there are also groups in which decreased use of one type of substance is associated with increased use of another.

Another further exploration of the data would be to study changing patterns across time. Short-term changes immediately after the measures came into effect may differ from long-term changes after months of restrictions and accumulating socio-economic consequences. In addition, government measures varied with infection rates over time. In fact, on 14 October 2020 (the day after survey data for this paper was extracted) a new “partial lockdown” was enforced, that has been tightened into a “hard lockdown” since 14 December 2020 (while the survey was still ongoing).

### Limitations

Because of anonymity, it cannot be ascertained that the sample consist of unique individuals. A selection was made of respondents who answered negatively to the question whether they had previously participated. The chance of duplication is estimated to be small because no incentives were given and there was nothing to gain by filling out the questionnaire for the second or subsequent time and lying about it.

This study cannot claim optimal generalizability due to under-coverage and self-selection inherent to web surveys (43). Substance use is over-represented in the total sample (tobacco 54.5%, alcohol 85.3%, cannabis 48.7%, other drugs 50.6%) when compared to the general Dutch population (tobacco 22.4%, alcohol 80.4%, cannabis 7.5%, ecstasy 2.8%, amphetamines 1.1%, cocaine 1.6%, nitrous oxide 2.7%) (44), and even subsamples of respondents who use these substances may not be representative of populations of users. Because of under-coverage some groups of users will be insufficiently presented in our sample (e.g., elderly or marginalized users), while self-selection may have caused our sample to be skewed toward young users who have experienced changing consumption patterns. Furthermore, the sample studied is relatively young (mainly 16–24 years). In this age group personality and brain development is still in process, and both are of significant influence on substance use trajectories (45). Proportion sizes of discontinued, decreased, unchanged, increased and (re)commenced use can therefore not be extrapolated to absolute figures for the general population.

To limit questionnaire length, detailed information on frequency and amount of use was not collected for drugs other than tobacco, alcohol and cannabis. Changing patterns in the use of other drugs is therefore based on self-report rather than objective measures. When asked if using more or less drugs than “pre-corona” the reference time frame was the “pre-corona” year (15 March 2019–15 March 2020), but respondents may have reflected on the time directly preceding the coronavirus measures. This period is “slow season” for music festivals (29–53 per month in Jan-Mar 2019; 118–174 per month in Apr-Sep 2019) (46), which are often preferred settings for drugs like ecstasy and nitrous oxide. Compared to that time, any drug use after the coronavirus measures came into effect could have felt like an increase in the respondents' minds. This may explain why respondents reported increased use of other drugs that could not be corroborated with increased prevalence rates. Measuring change in other drug use in a single question also impedes the ability to examine more complex patterns like selection (choosing a particular drug to use or quit) or substitution (replacing one drug with another).

Finally, some remarks about the definition of changing patterns. This was based on the frequency and amount of use in two relatively short periods for tobacco, alcohol and cannabis (“pre-corona” month and last week). Neither of these periods may have reflected “typical” consumption patterns and any absence of use may be “coincidental.” Discontinued use (defined as “pre-corona” use, but no last week use), for instance, may also include incidental (non-weekly) use and does not necessarily imply that there has not been any use since the coronavirus measures came into effect. Furthermore, increased and decreased use was derived from the difference in weekly consumption. In some cases differences were limited to only a few cigarettes/glasses/joints per week. For one person a small decrease in substance use may imply a clinically relevant risk level reduction, while for another a large decrease may not affect risk level outcome. For example, a female decreasing weekly consumption from 15 to 13 glasses will thereby fall below the threshold of excessive drinking, defined as more than 14 glasses a week for females in the Netherlands (44), while for a female decreasing weekly consumption from 25 to 15 glasses the end point will not fall below the threshold and the risk level outcome will remain that of excessive drinking. The current classification of changing patterns does not discern between these two examples and both are assigned to the “less” group. The aim of this paper was to explore different patterns of change in substance use. A more comprehensive examination of decreased or increased use may take different end points in terms of amount and frequency into account, but the survey did not measure any functional outcomes (e.g., health or use-related problems).

### Conclusion

People show varying changing patterns of substance use since social distancing and other measures combatting the coronavirus came into effect. Some are using more than “pre-corona,” some are using less, and others are currently not using at all. Especially the use of drugs like ecstasy and nitrous oxide was discontinued or decreased due to the lack of social occasions for use. Those who increased their intake of tobacco, alcohol, cannabis or other drugs are more likely to report coping motives for use.

## Data Availability Statement

The raw data supporting the conclusions of this article will be made available by the authors, without undue reservation.

## Ethics Statement

Ethical review and approval was not required for the study on human participants in accordance with the local legislation and institutional requirements. The patients/participants provided their written informed consent to participate in this study.

## Author Contributions

AB contributed to the study design, questionnaire development, analysis, interpretation, and wrote the manuscript. FB contributed to the study design, interpretation, and assisted in drafting and reviewing the manuscript. JN contributed to the study design, questionnaire development, coordination of data collection, and assisted in drafting and reviewing the manuscript. All authors agree to be accountable for the content of the work and provided approval of the manuscript.

## Conflict of Interest

AB is employed by the Amsterdam University of Applied Science, a public knowledge institution offering higher professional education. FB and JN are employed by Arkin, a non-profit organization for mental health and addiction care. The authors declare that this study was funded from an annual grant from the municipality of Amsterdam as part of a structural monitoring effort. The funder was not involved in the study design, collection, analysis, interpretation of data, the writing of this article or the decision to submit it for publication.
