# Supplementary material for: Changing Patterns of Substance Use During the Coronavirus Pandemic: Self-Reported Use of Tobacco, Alcohol, Cannabis, and Other Drugs
Source: Front Psychiatry. 2021 May 26;12:633551. doi: 10.3389/fpsyt.2021.633551 (PMC8187560; doi:10.3389/fpsyt.2021.633551)
Supplement: Supplementary file 1 [file Data_Sheet_1.docx]

# Supplementary material

*Table Suppl.1: Associations between demographic characteristics and changes in tobacco use*

|  | Stopped ^a^  (n=344) | Less ^b^  (n=762) | Same ^c^  (n=1,003) | More ^d^  (n=980) | Started ^e^  (n=221) | ChiSq / F (df) | p | Cramer’s V / EtaSq |
| --- | --- | --- | --- | --- | --- | --- | --- | --- |
| Sex |  |  |  |  |  |  |  |  |
| Male | 50.1% | 56.0% | 63.5% | 54.4% | 49.8% | 31.174 (4) | <.001 | .097 |
| Female | 49.9% | 44.0% | 36.5% | 45.6% | 50.2% |  |  |  |
| Age |  |  |  |  |  |  |  |  |
| Average  (SD) | 23.6  (12.1) | 20.9  (8.7) | 26.4  (14.6) | 22.3  (10.0) | 23.1  (11.4) | 27.748 (4) | <.001 | .032 |
| 16-17 | 29.1% | 30.2% | 19.3% | 28.3% | 32.6% | 123.949 (12) | <.001 | .112 |
| 18-24 | 48.8% | 58.9% | 52.1% | 53.9% | 47.1% |  |  |  |
| 25-39 | 12.8% | 6.6% | 12.7% | 10.0% | 13.6% |  |  |  |
| 40+ | 9.3% | 4.3% | 15.9% | 7.9% | 6.8% |  |  |  |
| Place of residence |  |  |  |  |  |  |  |  |
| Small (pop. < 100.000) | 48.0% | 53.7% | 57.7% | 50.5% | 47.5% | 17.744 (4) | .001 | .079 |
| Large (pop. > 100.000) | 52.0% | 46.3% | 42.3% | 49.5% | 52.5% |  |  |  |
| Student |  |  |  |  |  |  |  |  |
| No | 26.7% | 21.7% | 43.9% | 24.8% | 25.3% | 155.734 (8) | <.001 | .153 |
| Secondary (vocational) | 43.0% | 49.9% | 39.5% | 45.3% | 39.8% |  |  |  |
| Higher prof. or university | 30.2% | 28.5% | 16.7% | 29.9% | 34.8% |  |  |  |
| Work |  |  |  |  |  |  |  |  |
| Not working | 41.9% | 43.6% | 36.2% | 38.5% | 36.7% | 34.219 (8) | <.001 | .072 |
| Working from home | 12.2% | 6.2% | 8.7% | 11.7% | 14.0% |  |  |  |
| Working on location | 45.9% | 50.3% | 55.1% | 49.8% | 49.3% |  |  |  |
| Household |  |  |  |  |  |  |  |  |
| Alone | 9.2% | 6.4% | 12.7% | 9.6% | 12.5% | 45.601 (12) | <.001 | .069 |
| With partner/housemate(s) | 23.7% | 20.9% | 22.4% | 22.2% | 21.3% |  |  |  |
| With parent(s) | 60.1% | 68.9% | 56.6% | 62.2% | 57.9% |  |  |  |
| With child(ren) | 7.1% | 3.8% | 8.3% | 6.1% | 8.3% |  |  |  |

*Table Suppl.2: Associations between demographic characteristics and changes in alcohol use*

|  | Stopped ^a^  (n=585) | Less ^b^  (n=1,505) | Same ^c^  (n=1,098) | More ^d^  (n=1,661) | Started ^e^  (n=327) | ChiSq / F (df) | p | Cramer’s V / EtaSq |
| --- | --- | --- | --- | --- | --- | --- | --- | --- |
| Sex |  |  |  |  |  |  |  |  |
| Male | 41.2% | 51.9% | 55.5% | 51.1% | 46.6% | 33.768 (4) | <.001 | .081 |
| Female | 58.8% | 48.1% | 44.5% | 48.9% | 53.4% |  |  |  |
| Age |  |  |  |  |  |  |  |  |
| Average  (SD) | 27.7  (16.0) | 25.0  (13.3) | 35.0  (20.0) | 26.9  (14.3) | 25.5  (14.3) | 77.218 (4) | <.001 | .056 |
| 16-17 | 25.0% | 21.1% | 12.8% | 20.3% | 32.1% | 301.235 (12) | <.001 | .139 |
| 18-24 | 43.9% | 54.2% | 37.6% | 47.3% | 41.3% |  |  |  |
| 25-39 | 10.4% | 12.0% | 13.7% | 15.2% | 11.0% |  |  |  |
| 40+ | 20.7% | 12.7% | 36.0% | 17.2% | 15.6% |  |  |  |
| Place of residence |  |  |  |  |  |  |  |  |
| Small (pop. < 100.000) | 51.8% | 50.9% | 56.8% | 48.2% | 56.3% | 23.017 (4) | <.001 | .067 |
| Large (pop. > 100.000) | 48.2% | 49.1% | 43.2% | 51.8% | 43.7% |  |  |  |
| Student |  |  |  |  |  |  |  |  |
| No | 37.6% | 31.0% | 55.4% | 37.3% | 33.9% | 217.501 (8) | <.001 | .145 |
| Secondary (vocational) | 39.1% | 35.1% | 25.1% | 34.7% | 48.3% |  |  |  |
| Higher prof. or university | 23.2% | 33.9% | 19.5% | 28.1% | 17.7% |  |  |  |
| Work |  |  |  |  |  |  |  |  |
| Not working | 41.2% | 37.7% | 35.8% | 37.0% | 40.7% | 29.357 (8) | <.001 | .053 |
| Working from home | 15.6% | 12.0% | 17.1% | 17.4% | 12.8% |  |  |  |
| Working on location | 43.2% | 50.3% | 47.1% | 45.6% | 46.5% |  |  |  |
| Household |  |  |  |  |  |  |  |  |
| Alone | 14.6% | 10.3% | 15.3% | 10.8% | 12.5% | 145.138 (12) | <.001 | .098 |
| With partner/housemate(s) | 22.7% | 26.7% | 29.3% | 27.0% | 15.9% |  |  |  |
| With parent(s) | 53.3% | 56.0% | 38.5% | 50.8% | 61.7% |  |  |  |
| With child(ren) | 9.3% | 7.0% | 17.0% | 11.4% | 10.0% |  |  |  |

*Table Suppl.3: Associations between demographic characteristics and changes in cannabis use*

|  | Stopped ^a^  (n=483) | Less ^b^  (n=514) | Same ^c^  (n=659) | More ^d^  (n=973) | Started ^e^  (n=327) | ChiSq / F (df) | p | Cramer’s V / EtaSq |
| --- | --- | --- | --- | --- | --- | --- | --- | --- |
| Sex |  |  |  |  |  |  |  |  |
| Male | 51.6% | 70.6% | 71.7% | 65.4% | 42.2% | 123.911 (4) | <.001 | .205 |
| Female | 48.4% | 29.4% | 28.3% | 34.6% | 57.8% |  |  |  |
| Age |  |  |  |  |  |  |  |  |
| Average  (SD) | 20.6  (7.2) | 19.3  (4.7) | 22.2  (10.4) | 19.8  (5.8) | 20.6  (6.9) | 14.235 (4) | <.001 | .019 |
| 16-17 | 35.4% | 35.0% | 25.2% | 30.1% | 27.8% | 70.875 (12) | <.001 | .089 |
| 18-24 | 52.0% | 59.3% | 58.3% | 62.4% | 60.2% |  |  |  |
| 25-39 | 8.5% | 4.5% | 11.1% | 5.8% | 9.5% |  |  |  |
| 40+ | 4.1% | 1.2% | 5.5% | 1.7% | 2.4% |  |  |  |
| Place of residence |  |  |  |  |  |  |  |  |
| Small (pop. < 100.000) | 53.2% | 55.6% | 51.9% | 50.9% | 44.0% | 11.579 (4) | .021 | .063 |
| Large (pop. > 100.000) | 46.8% | 44.4% | 48.1% | 49.1% | 56.0% |  |  |  |
| Student |  |  |  |  |  |  |  |  |
| No | 20.1% | 18.3% | 31.3% | 19.0% | 16.8% | 64.856 (8) | <.001 | .105 |
| Secondary (vocational) | 48.0% | 55.6% | 45.2% | 48.4% | 46.5% |  |  |  |
| Higher prof. or university | 31.9% | 26.1% | 23.5% | 32.6% | 36.7% |  |  |  |
| Work |  |  |  |  |  |  |  |  |
| Not working | 36.2% | 39.5% | 38.7% | 43.6% | 42.2% | 13.583 (8) | .093 | .048 |
| Working from home | 9.3% | 6.6% | 7.1% | 6.1% | 8.6% |  |  |  |
| Working on location | 54.5% | 53.9% | 54.2% | 50.4% | 49.2% |  |  |  |
| Household |  |  |  |  |  |  |  |  |
| Alone | 8.0% | 5.4% | 10.2% | 6.2% | 5.9% | 27.750 (12) | .006 | .057 |
| With partner/housemate(s) | 19.1% | 18.3% | 20.0% | 20.0% | 24.0% |  |  |  |
| With parent(s) | 68.0% | 73.5% | 66.7% | 70.8% | 64.5% |  |  |  |
| With child(ren) | 4.9% | 2.8% | 3.1% | 2.9% | 5.6% |  |  |  |

*Table Suppl.4: Associations between demographic characteristics and changes in other drug use ^f^*

|  | Stopped ^a^  (n=935) | Less ^b^  (n=637) | Same ^c^  (n=532) | More ^d^  (n=778) | Started ^e^  (n=190) | ChiSq / F (df) | p | Cramer’s V / EtaSq |
| --- | --- | --- | --- | --- | --- | --- | --- | --- |
| Sex |  |  |  |  |  |  |  |  |
| Male | 56.3% | 61.7% | 62.9% | 57.8% | 53.7% | 10.669 (4) | .030 | .059 |
| Female | 43.7% | 38.3% | 37.1% | 42.2% | 46.3% |  |  |  |
| Age |  |  |  |  |  |  |  |  |
| Average  (SD) | 21.8  (7.2) | 23.2  (8.6) | 22.3  (8.7) | 21.3  (7.7) | 19.4  (6.7) | 10.441 (4) | <.001 | .013 |
| 16-17 | 22.0% | 14.3% | 18.8% | 16.5% | 46.3% | 134.101 (12) | <.001 | .121 |
| 18-24 | 58.8% | 62.2% | 62.4% | 68.6% | 46.3% |  |  |  |
| 25-39 | 15.0% | 16.8% | 13.3% | 12.6% | 4.7% |  |  |  |
| 40+ | 4.2% | 6.8% | 5.5% | 2.3% | 2.6% |  |  |  |
| Place of residence |  |  |  |  |  |  |  |  |
| Small (pop. < 100.000) | 49.1% | 47.9% | 42.7% | 46.3% | 51.1% | 7.238 (4) | .124 | .049 |
| Large (pop. > 100.000) | 50.9% | 52.1% | 57.3% | 53.7% | 48.9% |  |  |  |
| Student |  |  |  |  |  |  |  |  |
| No | 27.9% | 33.0% | 32.3% | 27.2% | 16.8% | 56.296 (8) | <.001 | .096 |
| Secondary (vocational) | 36.7% | 31.4% | 34.2% | 38.2% | 59.5% |  |  |  |
| Higher prof. or university | 35.4% | 35.6% | 33.5% | 34.6% | 23.7% |  |  |  |
| Work |  |  |  |  |  |  |  |  |
| Not working | 37.6% | 35.9% | 37.8% | 37.5% | 49.5% | 30.556 (8) | <.001 | .071 |
| Working from home | 12.2% | 14.6% | 10.5% | 8.1% | 5.8% |  |  |  |
| Working on location | 50.2% | 49.5% | 51.7% | 54.4% | 44.7% |  |  |  |
| Household |  |  |  |  |  |  |  |  |
| Alone | 8.5% | 11.7% | 8.0% | 9.8% | 5.3% | 31.174 (12) | .002 | .059 |
| With partner/housemate(s) | 24.4% | 28.4% | 31.1% | 27.8% | 18.7% |  |  |  |
| With parent(s) | 61.9% | 55.0% | 55.8% | 58.9% | 70.1% |  |  |  |
| With child(ren) | 5.2% | 5.0% | 5.1% | 3.5% | 5.9% |  |  |  |
